# How can graduate students’ research pressure be transformed into motivation and innovative behavior? The role of graduate students’ mentorship homegate (or team) support

**DOI:** 10.3389/fpsyg.2024.1439478

**Published:** 2025-01-08

**Authors:** Qiming Li, Xian Du, Hua Chen, Xianli Zhou

**Affiliations:** ^1^Psychological Research and Counseling Center, Southwest Jiaotong University, Chengdu, China; ^2^Graduate School, Southwest Jiaotong University, Chengdu, China

**Keywords:** challenging-hindering research pressure, graduate students’ mentorship homegate (or team) support, intrinsic research motivation, research role identity, innovative behavior

## Abstract

**Introduction:**

This study explores how graduate students’ mentorship homegate (or team) support (GSMTS) and challenging-hindering pressures impact their intrinsic motivation for research, identification with research roles, and innovative behaviors.

**Methods:**

Data from 548 graduate students were collected using convenience sampling and analyzed using Amos and SPSS statistical software package via questionnaires distributed to universities in SiChuan province of China.

**Result:**

The findings reveal that (1) research stress can not directly and positively predict innovative behaviors among graduate students, while intrinsic research motivation and research role identification mediate the relationship between research stress and graduate students’ innovative behavior; (2) hindering research pressure negatively impacts the intrinsic motivation for research, whereas challenging research pressure has a positive effect; (3) GSMTS directly fosters innovative behaviors among graduate students, with intrinsic motivation and roles’ identification for research as sequential mediators; and (4) GSMTS positively moderates the relationship between challenging research pressure and both the intrinsic motivation for research and role identity.

**Discussion:**

This suggests that higher education institutions should cultivate an optimal research and innovation environment for graduate students by increasing challenging research pressure and reducing hindering pressure. They should also emphasize the development of graduate students’ intrinsic motivation for research and identification with research roles. Concurrently, the role of GSMTS should be highlighted to facilitate both the direct and indirect development of graduate students’ innovative behaviors.

## Introduction

As global competition intensifies and a knowledge economy has emerged, fostering innovation among graduate students has become a focal point of postgraduate education. Chinese higher education institutions are continually elevating academic expectations for graduate students; yet a substantial shortfall exists in meeting the practical demands of society regarding innovative thought, behavior, and output (Wang and Sun, 2022). Consequently, fostering creativity in graduate students is a formidable challenge, as they face escalating academic and research pressures. For example, graduate students are expected to engage in research projects and publish papers, with publication often linked to academic rewards and graduation requirements. Many universities explicitly mandate a certain number of publications for graduation (Zhao et al., 2024), which creates tremendous academic pressure and anxiety among students. Additionally, these publications invoke rigorous double-blind reviews, markedly increasing research pressure (Li and Li, 2023). Previous research indicates that research pressure can create divergent outcomes, either stimulating intrinsic motivation and enhancing research output or, conversely, resulting in psychological health issues and academic misconduct (Li et al., 2018). Thus, transforming research pressure into intrinsic research motivation and innovative behavior has become a focal point of attention in graduate education.

## Literature review

### Relationship between challenging-hindrance research pressure and innovative behavior

Graduate students’ innovative behavior primarily refers to the process by which students not only apply their specialized theoretical knowledge, guided by innovative consciousness and creative thinking, to solve problems in novel ways but also place creative thought into practice to achieve innovative results (Zhang et al., 2022). The relationship between stress and innovative behavior has always been a research focus but is highly controversial, and most studies have focused on corporate employees. Although stress can promote innovative behavior (Sun et al., 2018), it can also affect it negatively (Cao et al., 2021). As these contrasting findings may be due to the lack of differentiation in previous studies, this study further explores the relationship between challenging-hindrance research pressure and innovative behavior.

Research pressure on graduate students can be divided into challenging and hindering pressures. Challenging research pressures are characterized by heavy research tasks, high standards for research innovation, and urgency; whereas hindering research pressures manifest as a scarcity of learning resources and an unclear and unfair distribution of research tasks by mentors (Liu, 2017). Empirical studies from the perspectives of social exchange, social cognition, and the conservation of resources (Chen et al., 2021) have demonstrated that challenging pressure can facilitate innovative employee behavior, whereas hindering pressure can inhibit it (Peng, 2021; Xu et al., 2021). Specifically, employees facing challenging pressures may perceive opportunities for personal growth or development (Du et al., 2014), which can ignite their enthusiasm to overcome these pressures, and consequently, they exhibit innovative behavior. This suggests that graduate students facing challenging research pressure may be more inclined to proactively seek novel solutions to problems, potentially benefiting their innovative behavior; however, graduate students confronted with hindering pressure may respond to research tasks passively and negatively, which could adversely affect their innovation.

Accordingly, this study proposes Hypothesis 1a (H1a): Challenging research pressure positively predicts graduate students’ innovative behavior, whereas hindering research pressure negatively predicts graduate students’ innovative behavior.

### Mediating role of intrinsic research motivation and research role identity

Intrinsic research motivation refers to graduate students’ internal, spontaneous, and enduring passion and commitment to research activities (Dong et al., 2021). According to the conservation of resources theory, graduate students may be inspired to invest more resources in future research activities due to the sense of academic achievement gained from completing challenging research tasks. In contrast, graduate students who face hindering research pressure may perceive these pressures as insurmountable, reducing their passion for research and leading them to adopt a resource-conservation approach (Zhang et al., 2018). Empirical studies have also revealed that hindering research pressure can weaken graduate students’ research motivation (Van den Broeck et al., 2010). This suggests that differences may exist in the relationship between challenging-hindering research pressures and graduate students’ intrinsic research motivation.

Additionally, intrinsic motivation is a crucial internal factor for graduate students to engage in innovative behavior (Cheng et al., 2013), encouraging them to actively participate in research and achieve more with less effort. The self-determination theory emphasizes intrinsic motivation’ s influence on behavior (Deci et al., 1999). Further, Amabile and Pratt (2016) considered the dynamic componential theory of creativity to further emphasize that innovative behavior depends on the synergy between individual intrinsic motivation and the external environment. Meta-analyses indicate a significant, positive relationship between intrinsic motivation and creative performance (Cerasoli et al., 2014; de Jesus et al., 2013; Liu et al., 2016). Therefore, a close relationship may exist between challenging-hindering scientific research pressures and graduate students’ intrinsic motivation and innovative behavior in research.

Identifying the research role involves graduate students incorporating research into their self-concept (Yin et al., 2016). Scholars have found that challenging research pressures can enhance graduate identification, whereas hindering pressures can reduce it (Li et al., 2018b). The role identity theory posits that aligning individual behavior with expected social roles is central to role identification (Albert et al., 2000; Wang, 2010). This implies that once graduate students identify with a research role, they may be driven to engage in innovative activities. Previous research also indicates that research role identification positively influences creativity and resilience (Perez et al., 2014), which are foundational for innovative behavior. Therefore, research role identification likely has a close relationship with innovative behavior.

Moreover, challenging and hindering scientific research pressures may differently affect the relationships among intrinsic motivation, research role identification, and innovative behavior. Studies in corporate settings suggest that while challenging pressures can boost self-efficacy and encourage innovation, obstructive pressures might lower self-efficacy and dampen creativity (An et al., 2021). Self-efficacy not only propels motivation but also forms the basis of research role identification. Moreover, intrinsic motivation is foundational to research role identification, as it drives graduate students’ passion for and engagement in scientific activities, facilitating the recognition of their researcher identity in the academia (Zhao and Jia, 2022).

Therefore, this study proposes Hypotheses 2a–2c (H2a-H2c): Hypothesis 2a posits that internal research motivation mediates the relationship between research pressure and the innovative behavior of graduate students. Hypothesis 2b states that research role identity mediates the relationship between research pressure and graduate students’ innovative behavior. Finally, Hypothesis 2c proposes that both internal research motivation and research role identity sequentially mediate the relationship between research pressure and the innovative behavior of graduate students.

### Role of graduate students’ mentorship homegate (or team) support

According to Eisenberger and Huntington’s (1986) organizational support theory, graduate students’ mentorship homegate (or team) support (GSMTS) is the assistance and support those graduate students receive from their mentors or peers during their academic and research endeavors. The “mentor’s gate” is an important venue where graduate mentors and students interact and communicate, thereby educating and influencing students. It is significant in the graduate education process (Liu, 2021). Graduate students primarily conduct academic exchange activities within graduate students’ mentorship homegate. The academic discussions, intellectual meetings, and emotional exchanges between mentors and graduate students, as well as among fellow students, are key influencing factors for research innovation behavior. For instance, Hu (2017) suggested that mentors and peers are the closest contacts for graduate students within the university, significantly impacting their learning and research activities. Further, graduate students’ innovative behavior results from the interplay of external and internal factors (Wu et al., 2014), with GSMTS being a significant external influence. Hou et al. (2016) observed that autonomy support from mentors can effectively predict and enhance graduate students’ innovative behavior. Therefore, GSMTS may directly foster innovation among graduate students.

Moreover, such support closely connects to graduate students’ intrinsic motivation and identification with their research. GSMTS fosters a sense of belonging while encouraging a proactive approach to research challenges (Zhu et al., 2016). This sense of belonging is a key pathway to forming a research role identity; being proactive is a significant manifestation of intrinsic research motivation, suggesting that GSMTS can influence both. In university research activities, graduate students’ interactions with mentors and peers within their research team and the support they receive can further promote their self-identification as researchers and their active participation in research activities (Li et al., 2018).

Additionally, GSMTS may further modulate the relationship between scientific research pressure and innovative behavior, intrinsic motivation, and research role identification. Studies on employees indicate that challenging work pressures, moderated by perceived organizational support, enhance proactive innovative behaviors (Yang et al., 2019), with organizational support acting as a buffer in stressful situations (Zhang, 2020). Hence, perceived organizational support moderates the relationship between stress and innovative behavior (Janssen, 2000; Du et al., 2014); subsequently, GSMTS could also buffer graduate students’ research pressure. Moreover, educators’ support has been shown to predict students’ motivation and academic performance (Ahmed et al., 2018), indicating that research pressure might boost graduate students’ intrinsic motivation and strengthen research role identification through the moderation of GSMTS.

Thus, this study proposes Hypotheses 1b (H1b): GSMTS has a positive predictive effect on graduate students’ innovative behavior. Further, Hypothesis 3 (H3) states that this also positively moderates the relationship between scientific research pressure and graduate students’ innovative behavior, intrinsic motivation, and research role identification.

## Research methods

### Participants

A questionnaire survey was administered utilizing both group and individual methodologies among graduate students. A total of 548 graduate students from various universities in Sichuan Province participated in the survey, with all participants having been enrolled for a minimum duration of 6 months. The gender distribution was as follows: 275 males (50.2%), 267 females (48.7%), and 6 missing (1.1%). The grade distribution was as follows: first-year (251 students, 45.8%), second-year (158, 28.8%), and third-year and above (138, 25.2%), with 1 missing student (0.2%). The distribution by academic discipline was as follows: humanities and social sciences (201 students, 36.7%), science (87, 15.9%), engineering (256, 46.7%), and medicine (3, 0.5%), with 1 missing (0.2%). The distribution by degree type was professional degrees (212 students, 38.7%) and academic degrees (335, 61.1%), with 1 missing (0.2%).

### Survey questionnaire

The challenging-hindering scientific research pressure scale has been revised based on the scale developed by Yao and Ma (2021). It includes five items on challenging research pressure, such as undertaking challenging tasks, heavy research responsibilities, mastering numerous research methods, feeling time-pressured, and having a high volume of research tasks. The obstructive research pressure subscale also includes five items: unclear task allocation, unfair resource distribution, vague evaluation standards, cumbersome processes, and a sense of stagnation in one’ s academic career. The questionnaire consists of 10 items scored on a five-point scale, with responses ranging from 1 (completely disagree) to 5 (completely agree).

The administered questionnaire underwent an exploratory factor analysis, retaining 10 items with factor loadings between 0.66 and 0.87. These were aggregated into two factors: the first dimension, or “hindering research tasks,” accounted for 41.77% of the variance, and the second dimension, “challenging research tasks,” accounted for 20.02%. The total explained variance of the questionnaire was 61.79%, as presented in Table 1. Scores were calculated by summing the items within each dimension, with higher scores indicating greater research pressure experienced by the graduate students. The mean and standard deviation of the scale scores are shown in Table 2.

**Table 1:** Exploratory factor structure of challenging and hindering research pressure.

Questionnaire entries	Factor load	
	Challenging research pressure	Hindering research pressure
1. I am in the research group and need to undertake challenging scientific research tasks.	0.79	
2. I am in the research group and need to take on a heavy research responsibility.	0.84	
3. To engage in scientific research work, I need to master many research methods.	0.58	
4. In scientific research work, I often feel that time is tight.	0.66	
5. I have a large amount of research tasks to complete.	0.70	
6. In my research group, the allocation of scientific research tasks is unclear.		0.83
7. My research group has an unfair allocation of scientific research resources.		0.87
8. The evaluation criteria for the scientific research work I am engaged in are vague.		0.87
9. My academic career seems to have come to a standstill.		0.74
10. In the process of conducting scientific research work, I must go through cumbersome procedures.		0.79

**Table 2:** Descriptive statistics and correlation analysis of various research variables.

	*M* ± *SD*	1	2	3	4	5	6
1. Challenging research pressure	3.11 ± 0.71	1					
2. Hindering research pressure	2.37 ± 0.85	0.32^**^	1				
3. GSMTS	3.83 ± 0.64	−0.01	−0.41^**^	1			
4. Intrinsic motivation in scientific research	2.97 ± 0.93	0.23^**^	−0.14^**^	0.25^**^	1		
5. Identification of scientific research roles	2.56 ± 0.91	0.21^**^	−0.12^**^	0.18^**^	0.72^**^	1	
6. Innovation behavior of graduate students	3.28 ± 0.67	0.16^**^	−0.15^**^	0.27^**^	0.56^**^	0.48^**^	1

^*^p < 0.05; ^**^p < 0.01.

The GSMTS questionnaire was adapted from Devine and Hunter (2016) organizational support questionnaire and was culturally revised to focus on the GSMTS experienced by graduate students. Items such as When I encounter problems, my mentor group provides help and My mentor group values my goals and beliefs were included. The questionnaire consists of six items scored on a five-point scale, ranging from 1 (completely disagree) to 5 (completely agree). After administering the questionnaire, an exploratory factor analysis was conducted on the valid responses, retaining six items that loaded onto one factor with loadings between 0.74 and 0.85, explaining 65.06% of the variance, as presented in Table 3. The total score was obtained by summing the item scores, and reflects the level of support perceived by the graduate students, with higher scores indicating a greater sense of support. The mean and standard deviation of the scale scores are shown in Table 2.

**Table 3:** Exploratory factor structure of graduate students’ mentorship homegate (or team) support.

Questionnaire entries	Factor load
1. My mentorship homegate (or team) cares about my opinions.	0.74
2. My mentorship homegate (or team) is concerned about my mental health.	0.81
3. My mentorship homegate (or team) values my goals and values.	0.85
4. When I encounter problems, my mentorship homegate (or team) can provide assistance.	0.84
5. My mentorship homegate (or team) will forgive my unintentional mistakes.	0.80
6. If I need special assistance, my mentorship homegate (or team) is willing to assist me.	0.79

Zhang and Bartol (2010) developed a questionnaire on graduate students’ intrinsic motivation for scientific research that included a five-item research-related intrinsic motivation scale (e.g., “I am engaged in the pursuit of resolving intricate scientific research challenges”). They adopted a five-point scoring system, with responses ranging from 1 (completely disagree) to 5 (completely agree). The higher the score, the greater the intrinsic motivation for scientific research. The mean and standard deviation of the scale scores are shown in Table 2.

This study also adopted the research role identity scale developed by Robnett et al. (2015), which exhibits good reliability and validity in the domestic research population (Yin et al., 2016). This questionnaire consists of five items (e.g., “I am innately disposed to the vocation of scientific research”), with a five-point scoring system and responses ranging from 1 (completely disagree) to 5 (completely agree). The higher the score, the greater the graduate student’ s recognition of their research role. The mean and standard deviation of the scale scores are shown in Table 2.

Su et al. (2021) considered the innovation behavior scale developed by Scott and Bruce (1994) to make corresponding revisions to measure graduate students’ innovation behavior; the resulting adapted scale exhibits good reliability and validity. This questionnaire includes eight items (e.g., “I consistently contribute innovative ideas and concepts to my research endeavors”). And adopts a five-point scoring method, with responses ranging from 1 (completely disagree) to 5 (completely agree). The higher the score, the greater the impact on graduate students’ innovation behavior. The mean and standard deviation of the scale scores are shown in Table 2.

### Data analysis

The relevant data analysis and processing were completed using SPSS 26.0 and AMOS 17.0 statistical software.

## Results

### Construct validity and reliability

The structural validity of the five scales is presented in Table 4. The results of the confirmatory factor analysis (CFA) for the Challenging-Obstructive Research Pressure Scale indicate a good model fit (*χ^2^* = 106.20, *df* = 30, *p* < 0.001, CFI = 0.95, TLI = 0.97, RMSEA = 0.068), with factor loadings ranging from 0.34 to 0.94. The Cronbach’s *α* coefficients for the two factors are 0.78 (Challenging Research Pressure) and 0.89 (Hindering Research Pressure).

**Table 4:** Confirmatory factor analysis results for the scales.

		*α*	*χ*^2^	*χ^2^*/*df*	*p*	*df*	CFI	TLI	RMSEA
Scientific Research Pressure Scale	Challenging	0.78	106.20	3.54	0.000	30	0.97	0.95	0.068
	Hindering	0.89							
GSMTS Scale		0.89	27.99	4.66	0.000	6	0.99	0.97	0.082
Intrinsic Motivation for Scientific Scale		0.90	16.74	5.58	0.000	3	0.99	0.97	0.091
Research Role Identity Scale		0.94	27.28	5.46	0.000	5	0.99	0.98	0.090
Innovation Behavior Scale		0.90	90.42	5.02	0.000	18	0.97	0.95	0.086

^*^p < 0.05; ^**^p < 0.01.

The CFA results for the Graduate Students’ Mentorship Homegate Support Scale (χ^2^ = 27.99, df = 6, *p* < 0.001, CFI = 0.97, TLI = 0.99, RMSEA = 0.082) showed that the data fit of the model was acceptable, with factor loadings ranging from 0.54 to 0.84. The Cronbach’s α coefficient for this scale is 0.89.

For the Intrinsic Research Motivation Scale, the CFA results (*χ^2^* = 16.74, *df* = 3, *p* < 0.001, CFI = 0.97, TLI = 0.99, RMSEA = 0.091) show an acceptable data fit. Factor loadings range from 0.48 to 0.93, with a Cronbach’s α coefficient of 0.90, indicating good internal consistency.

The CFA results for the Research Role Identity Scale (*χ^2^* = 27.28, *df* = 5, *p* < 0.001, CFI = 0.98, TLI = 0.99, RMSEA = 0.090) show an acceptable data fit. Factor loadings range from 0.48 to 0.93, with a Cronbach’s α coefficient of 0.94, indicating good internal consistency.

The CFA results for the Innovative Behavior Scale (*χ^2^* = 90.42, *df* = 18, *p* < 0.001, CFI = 0.95, TLI = 0.97, RMSEA = 0.086) show an acceptable data fit. Factor loadings range from 0.65 to 0.80, with a Cronbach’s α coefficient of 0.90, indicating good internal consistency.

### Common method bias (CMB)

The present study employed Harman’s single-factor test to assess the presence of common method bias. The results revealed six common factors with eigenvalues greater than 1, with the primary common factor accounting for 28.75% of the variance. This percentage falls below the critical threshold of 40%, indicating that there is no significant common method bias present in this study.

### Descriptive statistics and correlation analysis of each research variable

Descriptive statistics and correlational analyses were conducted on the variables, as presented in Table 2. The results indicate significant correlations among the challenging scientific research pressure, hindering scientific research pressure, intrinsic motivation for research, research role identification, and graduate students’ innovative behavior (*p* < 0.01). GSMTS did not significantly correlate with challenging scientific research pressure (*p* > 0.05) but did significantly correlate with all other research variables (*p* < 0.01).

### Mediation analysis

**Figure 1:**
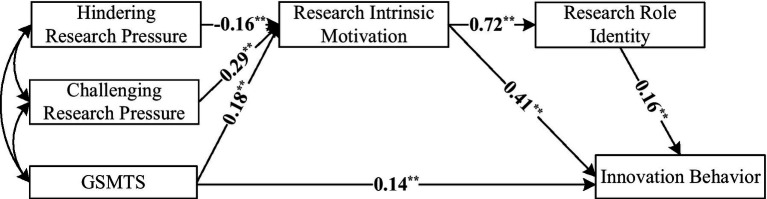
Mediation effect model (M1).

Challenging-hindering research stress and GSMTS are independent variables, with research motivation and role identification as mediators and graduate innovation as the dependent variable. As shown in Figure 1, the data fit was acceptable (*χ^2^*/*df =* 3.34, GFI = 0.98, AGFI = 0.96, NFI = 0.98, CFI = 0.98, and RMSEA = 0.07).

The SEM results showed that hindering research pressure had a weakly negative association with intrinsic research motivation (*β* = −0.16, *p* < 0.05), with a small effect size. GSMTS was positively associated with intrinsic research motivation (*β* = 0.18, *p* < 0.05), graduate students’ innovative behavior (*β* = 0.14, *p* < 0.05), research role identity and graduate students’ innovative behavior (*β* = 0.16, *p* < 0.05), with small effect sizes. Challenging research pressure had significantly positive association with intrinsic research motivation (*β* = 0.29, *p* < 0.05), intrinsic research motivation and research role identity (*β* = 0.72, *p* < 0.05), and graduate students’ innovative behavior (*β* = 0.41, *p* < 0.05), with moderate to large effect sizes.

Bootstrapping (*N* = 2,000) was used to test the significance of each path in the model, and Table 5 presents the results. All the model paths have confidence intervals that do not include zero, indicating significance, and the model’ s total effect size is 0.38. Hence, GSMTS directly and positively predicts graduate students’ innovative behavior. Additionally, both challenging-hindering research stress and GSMTS indirectly predict graduate students’ innovative behavior through the mediating variable of intrinsic research motivation. Intrinsic research motivation and research role identification also serve as chained mediators between challenging-hindrance research stress, GSMTS, and graduate students’ innovative behavior.

**Table 5:** Bootstrap analysis of the significance test of path effects.

Path	Estimated value of standardized effects	95% confidence interval	
		lower limit	upper limit
Hindering research pressure → intrinsic motivation in research → innovative behavior of graduate students	0.16 × 0.41 = 0.07	−0.08	−0.002
Hindering research pressure → intrinsic motivation for research → identification of research roles → innovative behavior of graduate students	0.16×0.72×0.16 = 0.02	−0.05	−0.02
Challenging research pressure → intrinsic motivation in research → innovative behavior of graduate students	0.29 × 0.41 = 0.12	0.06	0.09
Challenging research pressure → intrinsic motivation for research → identification of research roles → innovative behavior of graduate students	0.29 × 0.72 × 0.16 = 0.03	0.01	0.07
GSMTS → Graduate student innovation behavior	0.14	0.07	0.22
GSMTS → Intrinsic motivation in scientific research → Innovative behavior of graduate students	0.18 × 0.41 = 0.07	0.03	0.14
GSMTS → intrinsic motivation for scientific research → identification of scientific research roles → innovative behavior of graduate students	0.18 × 0.72 × 0.16 = 0.02	0.00	0.05

### Moderating analysis

A hierarchical regression was employed to assess the moderating role of GSMTS in the relationships between challenging-hindrance research stress and intrinsic research motivation, research role identification, and graduate students’ innovative behavior. First, independent t-tests and analysis of variance (ANOVA) were conducted to examine significant differences in innovative behavior across different academic majors and years, while controlling for gender, academic year, and major. Second, hindering-challenging research pressure and mentor support were treated as independent variables, with innovative behavior serving as the dependent variable to evaluate the main effects. Finally, intrinsic research motivation and research role identity were considered as moderating variables to investigate their moderating effects. The interaction terms of hindrance research stress with intrinsic research motivation, challenging research stress with intrinsic research motivation, hindrance research stress with research role identification, challenging research stress with research role identification, hindrance research stress with graduate students’ innovative behavior, and challenging research stress with graduate students’ innovative behavior were tested for their moderating effects. Table 6 presents the outcomes.

**Table 6:** Stepwise regression analysis of research intrinsic motivation, research role identification, and graduate students’ innovative behavior.

Step	independent variable	Model a1	Model a2	Model a3	Model b1	Model b2	Model b3	Model c1	Model c2	Model c3
Step1	Gender	−0.10^*^	−0.12^*^	−0.12^*^	−0.09	−0.10^*^	−0.10^*^	−0.12^*^	−0.14^**^	−0.14^**^
	major	0.05	0.05	0.04	0.03	0.03	0.03	−0.03	−0.03	−0.03
	grade	0.06	0.01	0.01	0.11^*^	0.07	0.07	0.17^**^	0.14^**^	0.13^**^
Step 2	Challenging research pressure (T)		0.27^**^	0.26^**^		0.24^**^	0.23^**^		0.16^**^	0.16^**^
	Hindering research pressure (Z)		−0.16^**^	−0.15^**^		−0.17^**^	−0.16^**^		−0.12^*^	−0.12^*^
	GSMTS (S)		0.20^**^	0.21^**^		0.12^**^	0.13^**^		0.23^**^	0.24^**^
Step 3	S x T			0.10^*^			0.12^**^			0.05
	S x Z			−0.00			−0.01			−0.06
*ΔR^2^*	–	0.02	0.16	0.17	0.03	0.11	0.13	0.05	0.15	0.15
*F*	–	4.39^**^	16.31^**^	13.17^**^	5.19^**^	11.38^**^	9.54^**^	8.87^**^	15.71^**^	12.10^**^

^*^p < 0.05; **p < 0.01.

The interaction effect of GSMTS and challenging research stress significantly influences intrinsic research motivation (*β* = −0.10, *p* < 0.05) and notably increases the explained variance in intrinsic research motivation (*ΔR^2^* = 0.01, *p* < 0.01). The interaction effect also significantly affects research role identification (*β* = −0.12, *p* < 0.01), with a significant increase in the explained variance for research role identification (*ΔR^2^* = 0.02, *p* < 0.01). However, the moderating effect of GSMTS on the relationship between challenging research stress and graduate students’ innovative behavior was not significant (*p* > 0.05), nor was the moderating effect of GSMTS on the relationship between hindrance research stress and research intrinsic motivation, research role identification, and graduate students’ innovative behavior (*p* > 0.05).

**Figure 2:**
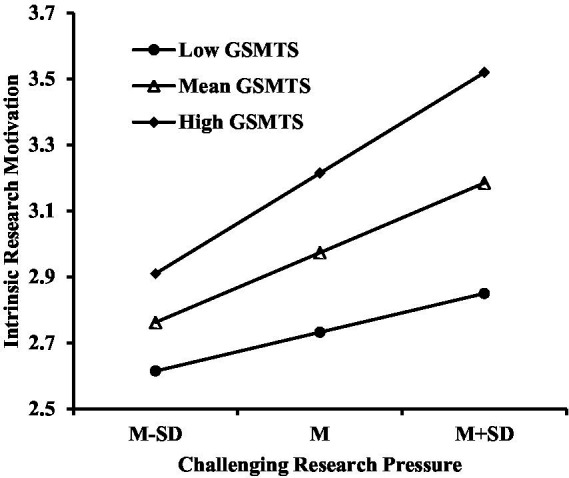
Moderation of challenging research pressure and intrinsic motivation by GSMTS.

Further analysis of simple slopes revealed that when GSMTS was one standard deviation below the mean, the predictive effect of challenging research pressure on intrinsic research motivation was significant (simple slope = 0.34, *t* = 6.26, *p* < 0.01). Similarly, when GSMTS was one standard deviation above the mean, the predictive effect of challenging research pressure on intrinsic research motivation remained significant (simple slope = 0.53, *t* = 3.80, *p* < 0.001), as shown in Figure 2.

**Figure 3:**
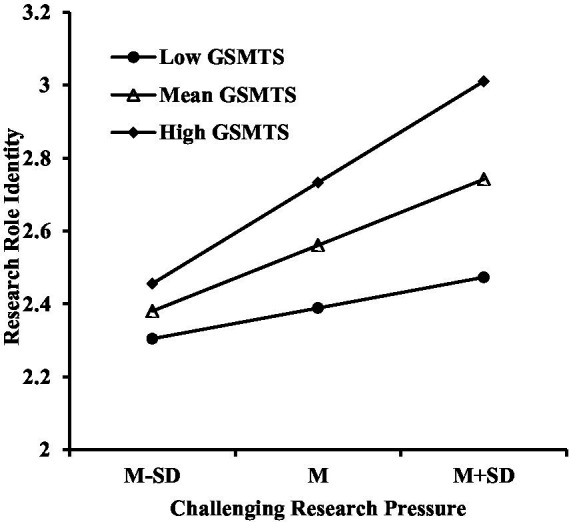
Moderation of challenging research pressure and research role identification by GSMTS.

Further analysis of simple slopes revealed that when GSMTS was one standard deviation below the mean, the predictive effect of challenging research pressure on research role identity was significant (simple slope = 0.29, *t* = 5.28, *p* < 0.01). Conversely, when GSMTS was one standard deviation above the mean, the predictive effect of challenging research pressure on research role identity was also significant (simple slope = 0.48, *t* = 3.48, *p* < 0.01), as shown in Figure 3.

Figures 2, 3 demonstrate that within the high GSMTS group, a linear upward trend occurs in graduate students’ intrinsic research motivation and research role identification as challenging research stress increases. In contrast, within the low GSMTS group, the increase in challenging research stress results in a more gradual change in intrinsic research motivation and research role identification.

## Discussion

This study constructed a moderated mediation model to confirm the chained mediating role of intrinsic research motivation and research role identification between research stress, GSMTS, and graduate innovative behavior. This confirmed the positive effect of GSMTS. The findings contribute to a deeper understanding of the mechanisms by which research stress affects graduate students’ innovative behavior and the conditions that enhance this mechanism.

### Intrinsic motivation and role identity as mediators in scientific research

This study revealed that challenging-hindrance research stress does not directly predict graduate students’ innovative behavior; thus, Hypothesis H1a was not supported. However, hindrance research stress has a significant, negative correlation with graduate students’ innovative behavior, whereas challenging research stress has a significant, positive correlation. This finding aligns with previous research indicating that challenging work stress can enhance employees’ innovative behavior, whereas hindering work stress can inhibit it (Cao et al., 2021). For employees, challenging work stress can be overcome by stimulating their coping ability and creative enthusiasm, whereas the negative impact of hindering work stress can prevent them from completing tasks (Zhao and Yang, 2020). For graduate students, however, both challenging and hindering research stress can cause significant psychological burdens. Although previous studies have demonstrated that challenging research stress can positively promote graduate students’ academic achievements (Du et al., 2019) and that hindrance research stress can negatively inhibit graduate students’ research performance (Liu, 2017), graduate students are just beginning their research activities, and their research foundation is relatively weak. Therefore, research stress may not directly affect their innovative behavior.

This study confirms that intrinsic research motivation and research role identification mediate the relationship between research stress and graduate students’ innovative behavior, supporting Hypothesis H2c. Specifically, intrinsic research motivation significantly mediates this relationship, thus supporting Hypothesis H2a; while Hypothesis H2b is not supported. Research role identification positively influences graduate innovation, consistent with previous research (Yin et al., 2016). Challenging research stress promotes innovation by enhancing intrinsic motivation and role identification, whereas hindrance stress inhibits innovation by diminishing these factors. Prior empirical studies have noted that challenging research stress positively affects graduate research performance by stimulating the achievement motivation (Wang et al., 2014), and hindrance stress exacerbates anxiety by reducing it (Yao and Ma, 2021). Intrinsic motivation is an active internal factor for graduate students, driving their engagement in research—and ultimately, innovation. Additionally, intrinsic motivation fosters research role identification, likely due to the internal and external recognition gained during research activities (Mantai, 2017). Thus, research stress indirectly impacts graduate innovation through the mediating roles of intrinsic motivation and research role identification.

### Direct and indirect roles of graduate students’ mentorship homegate (or team) support

This study revealed that GSMTS positively predicts graduate students’ innovative behaviors, confirming Hypothesis 1b. This finding aligns with previous research, which generally agrees that organizational support positively influences employees’ creativity (Hu, 2019; Huang et al., 2020). Graduate students’ innovative behaviors are inseparable from the support of their mentors and peers, whether by stimulating research ideas or providing guidance and suggestions throughout the research process. Both mentors and peers are significant, increasing the effectiveness of students’ research innovation; peer support enables graduate students to proactively face pressure and more actively address problems that arise in research innovation. However, the moderating role of GSMTS between research pressure and innovative behavior is not significant, possibly because as research pressure increases, GSMTS alone is insufficient to meet graduate students’ needs to successfully cope with research pressure.

GSMTS can significantly and positively modulate the relationship between challenging research pressure and both the intrinsic motivation for research and identification with the research role, partially confirming Hypothesis 3. Specifically, high GSMTS conditions demonstrate a more linear increase in graduate students’ intrinsic motivation for research and identification with the research role as challenging research pressure increases than low GSMTS. This may be because such support gives graduate students a sense of confidence and security when facing challenging research pressures; once these pressures are overcome, it enhances their intrinsic motivation for research and identification with the research role.

Previous empirical studies have also noted that support positively affects various research fields. For example, organizational support can positively predict employee performance (Wang and Chen, 2021), increase individuals’ motivation for development (Lin, 2022), and reduce individuals’ levels of anxiety and depression (Zhou et al., 2022). Thus, the positive role of GSMTS is significant in graduate students’ innovative behavior as well as their overall development. GSMTS indirectly influences graduate students’ innovative behavior by modulating the relationship between challenging research pressure and intrinsic motivation for research and identification with the research role. However, such support cannot modulate the relationship between hindering research pressure and intrinsic motivation for research or identification with the research role. This is possibly because obstructive research pressure leads to a greater loss of psychological resources and a more negative impact on graduate students, to the extent that it cannot be alleviated through GSMTS.

### Limitations

This study also has certain limitations. First, it did not employ a cross-sectional study design; thus, causality cannot be established. Future research could use longitudinal and experimental studies to further explore causal relationships. Second, this study did not integrate a variety of different methods due to various constraints, but the data was based on graduate students’ self-reports. In the future, more objective methods could be used to collect data. Last, the study was conducted only in the field of education and was limited to a specific geographical area, which restricts the results’ generalizability. Therefore, research conducted by other teams and processing data obtained at different times may lead to different outcomes.

### Implications

As the relationship between research pressure and graduate students’ innovative behavior indicates that stress is not entirely negative, our results offer the following implications. First, higher education institutions should establish appropriate graduate training models, create effective evaluation systems, and formulate related policies to support innovation training requirements, achieving a “top-down policy, bottom-up motivation” efficient transmission chain (Zhu and Zhou, 2018). Second, the mentor’ s role should be emphasized. Given challenging research pressure, mentors can provide graduate students with more research opportunities and tasks and demanding rigor while offering guidance; by allowing students to experience personal growth during the task completion process, this will enhance their intrinsic motivation for research and role identification. However, under obstructive research pressure, mentors should strive to provide learnable resources for graduate students, reduce unnecessary red tape, and allocate research tasks clearly and fairly, ensuring that students have sufficient interest and energy when participating in research activities.

The chain-mediating role of intrinsic motivation for research and identification with the research role between research pressure and innovative behavior suggests that intrinsic motivation profoundly affects whether graduate students engage in research and the type of results they wish to achieve. First, as guides on the research journey, mentors allocate research tasks with guidance and assistance, which endows graduate students with a sense of self-efficacy in research, thereby fostering their intrinsic motivation for research. This passion for research itself, when gradually transformed into innovative behavior and achieved research outcomes, promotes students’ identification with the researcher’ s identity. Identification with the research role will further motivate them to continuously engage in research activities, promoting the generation of innovative research outcomes and creating a virtuous cycle. Second, while an appropriate level of challenging research pressure is necessary, obstructive research pressure not only dampens the enthusiasm of graduate students for research but also damages their identification with the research role.

The positive correlation among GSMTS and graduate students’ innovative behavior, intrinsic motivation for research, and identification with the research role indicates that mentors and peers are essential sources of support for graduate students’ research. From the proposal of ideas to the determination and implementation of specific research content, mentors provide technical guidance as well as feedback on research revisions, which is undoubtedly crucial for graduate students. Therefore, mentors should encourage students to actively express their ideas and explore their interests, laying the groundwork for future innovative research. Additionally, mentors should learn to appreciate and recognize students’ inspiration, and peers should encourage and accept each other’ s views; this will facilitate students’ intrinsic motivation for research and identification with the research role. Thus, leveraging GSMTS alleviates research pressure on graduate students while directly or indirectly promoting their innovative behavior.

## Conclusion

The study offers the following conclusions. First, challenging research stress can not directly and positively foster graduate students’ innovative behavior, hindering research stress also fails to directly and negatively predict it. Nevertheless, both intrinsic motivation for research and identification with the research role serve as a chain of multiple mediators between research pressure and innovative behavior, this suggests that research stress indirectly impacts graduate innovation through the mediating roles of intrinsic motivation and research role identification. Second, challenging research pressure positively predicts the intrinsic motivation for research, whereas hindering research pressure negatively predicts it. Third, graduate students’ mentorship homegate support can directly and positively predict graduate students’ innovative behavior. Last, GSMTS positively modulates the relationship between challenging research pressure and intrinsic motivation for research, as well as between challenging research pressure and identification with the research role. Specifically, high GSMTS enhances the effect of challenging research pressure on intrinsic motivation for research and identification with the research role more than low GSMTS.

## Data Availability

The raw data supporting the conclusions of this article will be made available by the authors, without undue reservation.
